# Supplementary material for: A Likelihood Approach for Real-Time Calibration of Stochastic Compartmental Epidemic Models
Source: PLoS Comput Biol. 2017 Jan 17;13(1):e1005257. doi: 10.1371/journal.pcbi.1005257 (PMC5240920; doi:10.1371/journal.pcbi.1005257)
Supplement: S1 File — (TAR.GZ) [file pcbi.1005257.s014.tar.gz › HSPH_Online-SI-Revision/MSS10/n10-severe/n10-severe_NEW2_summary2.pdf]

peak -8	3w spec	3w cum	5w spec	5w cum	end	3w spec LOG	3w cum LOG	5w spec LOG	5w cum LOG	end LOG
mean	201.6	161.1	96.1	119.5	40.3	0.5	0.4	0.5	0.4	0.3
median	81.8	68.5	68.4	76.1	39.9	0.4	0.3	0.4	0.4	0.2
quantile .1	40.9	36.6	39.8	38.6	21.2	0.2	0.2	0.2	0.2	0.1
quantile .9	403.8	338.6	155.9	254.7	62.2	0.8	0.7	1.	0.2	0.6
39	38	35	38	27						

peak -4	3w spec	3w cum	5w spec	5w cum	end	3w spec LOG	3w cum LOG	5w spec LOG	5w cum LOG	end LOG
mean	31.1	28.4	39.2	27.8	39.9	0.2	0.1	0.2	0.2	0.2
median	28.4	24.	38.9	27.1	41.2	0.1	0.1	0.2	0.1	0.2
quantile .1	15.8	12.2	22.6	15.6	22.2	0.1	0.1	0.1	0.1	0.1
quantile .9	42.1	44.2	57.3	40.8	52.7	0.3	0.2	0.4	0.1	0.3
33	39	21	28	19						

peak	3w spec	3w cum	5w spec	5w cum	end	3w spec LOG	3w cum LOG	5w spec LOG	5w cum LOG	end LOG
mean	36.8	22.2	57.1	29.2	40.7	0.2	0.1	0.4	0.1	0.2
median	33.1	20.3	47.5	26.1	34.3	0.2	0.1	0.3	0.1	0.2
quantile .1	15.6	10.	20.6	10.5	13.1	0.1	0.	0.1	0.	0.1
quantile .9	62.4	38.2	99.3	49.8	69.9	0.4	0.2	0.8	0.	0.4
14	18	14	15	11						

peak +4	3w spec	3w cum	5w spec	5w cum	end	3w spec LOG	3w cum LOG	5w spec LOG	5w cum LOG	end LOG
mean	32.7	24.2	53.7	26.6	30.3	0.2	0.1	0.3	0.1	0.2
median	30.8	22.2	41.2	24.3	28.9	0.2	0.1	0.2	0.1	0.1
quantile .1	16.1	7.2	24.	8.9	14.3	0.1	0.	0.1	0.	0.1
quantile .9	51.9	41.6	83.8	46.6	46.2	0.3	0.2	0.6	0.	0.3
25	20	26	20	18						

peak +8	3w spec	3w cum	5w spec	5w cum	end	3w spec LOG	3w cum LOG	5w spec LOG	5w cum LOG	end LOG
mean	600.4	75.3	380.	82.5	88.6	0.6	0.3	0.9	0.3	0.4
median	68.7	44.	88.2	48.7	51.8	0.5	0.3	0.7	0.3	0.3
quantile .1	27.1	16.1	36.3	19.4	18.5	0.1	0.1	0.2	0.1	0.1
quantile .9	237.	130.5	718.	128.3	149.9	1.1	0.5	2.	0.1	0.8
26	18	28	19	21	{}	{}	{}	{}	{}	{}
